# Bidirectional causal relationships between the skin microbiome and psoriasis: Insights from Mendelian randomization analysis

**DOI:** 10.1097/MD.0000000000041736

**Published:** 2025-03-07

**Authors:** Shangyi Xu, Kaiwen Yang, Jin Qiu, Jianqiao Zhong, Dehai Xian

**Affiliations:** a Emergency Medicine Department, Luzhou People’s Hospital, Luzhou, China; b Anatomy Laboratory, Southwest Medical University, School of Basic Medicine, Luzhou, Cichuan, China.

**Keywords:** dysbiosis, Mendelian randomization, microbial features, psoriasis, skin microbiome

## Abstract

Psoriasis is a chronic inflammatory skin disease affecting 2% of the global population. Recent research suggests the skin microbiome plays a critical role in psoriasis. Skin microbiome data were obtained from the KORA FF4 study in Germany, and psoriasis data from FinnGen genome-wide association study summary statistics. Forward and reverse 2-sample Mendelian randomization (MR) analyses were conducted to assess causal relationships. Forward MR analysis identified several microbial features as risk factors for psoriasis, including the family Neisseriaceae in sebaceous skin (OR = 1.036, 95% CI: 1.010–1.062, *P* = .0054), ASV011 in dry skin (OR = 1.024, 95% CI: 1.000–1.048, *P* = .0490), and the order Clostridiales in moist skin (OR = 1.016, 95% CI: 1.000–1.032, *P* = .0449). Protective features included ASV016 (OR = 0.972, 95% CI: 0.949–0.994, *P* = .0136) and ASV053 (OR = 0.973, 95% CI: 0.954–0.992, *P* = .0054) in dry skin. Reverse MR analysis confirmed psoriasis as a significant risk factor for changes in the skin microbiome, with notable associations in the dry skin region for asv002 (OR = 1.266, 95% CI: 1.061–1.510, *P* = .027) and genus: Haemophilus (OR = 1.364, 95% CI: 1.065–1.746, *P* = .013). This study reveals bidirectional causal relationships between the skin microbiome and psoriasis, highlighting specific microbial features such as Neisseriaceae and Clostridiales as potential risk factors. Further research is needed to develop treatments that modulate the skin microbiome to improve psoriasis outcomes.

## 1. Introduction

Psoriasis is a chronic inflammatory skin disease affecting approximately 2% of the global population.^[1]^ It is characterized by red, scaly patches on the skin, significantly impacting the quality of life of affected individuals. The pathogenesis of psoriasis is multifactorial, involving genetic susceptibility, environmental factors, and immune system dysregulation.^[2,3]^

Recent research has shown that the skin microbiome plays a crucial role in the onset and progression of psoriasis.^[4]^ The skin microbiome consists of a collection of microorganisms living on the skin, which are essential for maintaining skin health and immune function.^[5]^ The skin microbiome of psoriasis patients differs significantly from that of healthy individuals, with changes in bacterial diversity and composition.^[6]^

A 20-year study in Japan found that infections are an important risk factor for psoriasis, with an incidence rate of 3.5% to 8.3%.^[7–9]^ Recent studies have emphasized the significant role of the microbiome in the development and exacerbation of psoriasis. Research indicates that the onset of psoriasis is associated with streptococcal infections.^[10]^ Although streptococcal infections are usually self-limiting, psoriasis can recur with infection recurrence.^[11]^ Dysbiosis of the skin microbiome is linked to psoriasis.^[12]^ Approximately 60% of psoriasis patients have Staphylococcus aureus colonization in lesional areas, compared to only 5% to 30% on normal skin. Furthermore, the lipophilic yeast Malassezia has been associated with the severity of psoriasis.^[13]^ In the skin of psoriasis patients, beneficial bacteria such as Propionibacterium and Lactobacillus are found to be reduced.^[14,15]^

To better understand the causal relationship between the skin microbiome and psoriasis, Mendelian randomization (MR) analysis has been introduced. MR is a method in epidemiology used to assess causal relationships between risk factors and disease outcomes.^[16]^ It simulates a randomized controlled trial by using genetic variants as instrumental variables, independent of the confounding factors that typically affect observational studies. This is a powerful tool because it provides evidence of causality rather than mere association, helping to identify potential intervention targets to improve health outcomes. Given the strong correlation between the skin microbiome and psoriasis, we employed the Mendelian Randomization method to evaluate the bidirectional causal effects between them, aiming to uncover potential causal links and provide direction for clinical treatment and research.

## 2. Materials and methods

### 2.1. Study design

Our study explores the bidirectional causal relationship between the skin microbiome and psoriasis using single nucleotide polymorphisms (SNPs) as instrumental variables (IVs). The validity of MR analysis relies on 3 core assumptions: the relevance assumption, which requires a strong association between the IV and the exposure; the independence assumption, which ensures that the IV is independent of confounding factors affecting both the exposure and the outcome; and the exclusion restriction assumption, which stipulates that the IV influences the outcome solely through the exposure.^[17]^ Through this rigorous study design, we aim to gain deeper insights into the role of the skin microbiome in the pathogenesis of psoriasis.

### 2.2. Skin microbiome data

The data for this study were sourced from an epidemiological study conducted in Germany, aimed at investigating the impact of cardiovascular diseases, metabolic diseases, and environmental factors on health. In the KORA FF4 study, approximately 1656 samples were collected.^[18]^ These samples included various skin microenvironments, such as dry environments (forearm), moist environments (antecubital fossa), and sebaceous gland-rich environments (forehead and retroauricular crease).

Skin samples were collected using standard operating procedures and analyzed using high-throughput 16S rRNA gene sequencing. Data processing was performed with the DADA2 tool for filtering and denoising, generating high-resolution operational taxonomic units (OTUs), and classification was annotated through the Ribosomal Database Project.

Genotype data were derived from genome-wide association studies (GWAS) and underwent quality control using the PLINK tool. Distance-based redundancy analysis and generalized linear models were used to assess associations between microbial features and genetic variations.

Meta-analyses were conducted using METAL and METASOFT tools, employing fixed-effect meta-analysis weighted by sample size.

Data processing and analysis primarily utilized R language and associated packages, including phyloseq, vegan, and mvabund. These steps ensured high data quality and reliability, laying a solid foundation for subsequent Mendelian randomization analysis.

### 2.3. Psoriasis data

Summary statistics for psoriasis were obtained from the FinnGen GWAS summary statistics (https://results.finngen.fi/en). FinnGen is a large-scale biobank research project that combines genomic data with health records to identify gene variants associated with diseases. This project integrates biobank samples and extensive health records from Finland. The study includes a large cohort of European ancestry, with 103,312 cases and 397,564 controls, accessible.

### 2.4. Selection of instrumental variables

We selected effective IVs based on 3 fundamental assumptions. First, IVs were identified according to the genome-wide significance threshold (*P* value < 5 × 10^−8^). Then, SNPs exhibiting linkage disequilibrium were excluded using criteria of *R*^2^ < 0.001 and Kb = 10,000. Different thresholds were set for various exposures to ensure a sufficient number of SNPs.^[19]^ Subsequently, PhenoScanner was used to exclude SNPs significantly associated with confounders, initially mitigating the impact of horizontal pleiotropy.^[20]^ To avoid bias from weak instruments, only IVs with an *F* statistic >10 were considered strong instruments. Finally, GWAS data from each skin microbiome dataset and psoriasis dataset were harmonized with the selected IVs.

### 2.5. Statistical analysis

**Figure 1.**
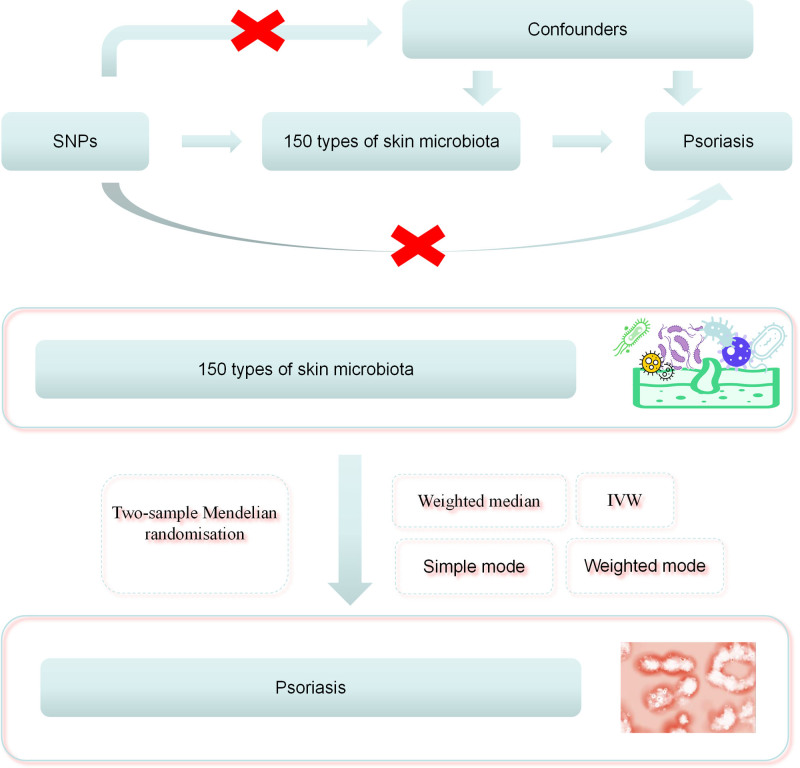
Schematic diagram illustrating the Mendelian randomization process.

We employed bidirectional 2-sample MR analysis to investigate the potential bidirectional causal effects of 150 skin microbiome features on psoriasis. The inverse variance weighted (IVW) method was used as the primary approach, with MR-Egger, weighted median, simple mode, and weighted mode as supplementary methods. Results were visualized using scatter plots. Sensitivity analyses were conducted to assess the robustness of the results. Cochran’s *Q* test and the *I*^2^ statistic were used to detect heterogeneity among the IVs. The MR-Egger intercept test was performed to evaluate the presence of horizontal pleiotropy.^[21]^ MR-PRESSO was further applied to examine potential horizontal pleiotropy.^[22]^ Additionally, leave-one-out sensitivity analysis was conducted to determine whether individual SNPs influenced the causal estimates. The Mendelian randomization analysis process is shown in Figure 1.

## 3. Results

### 3.1. Forward 2-sample Mendelian randomization study

**Figure 2.**
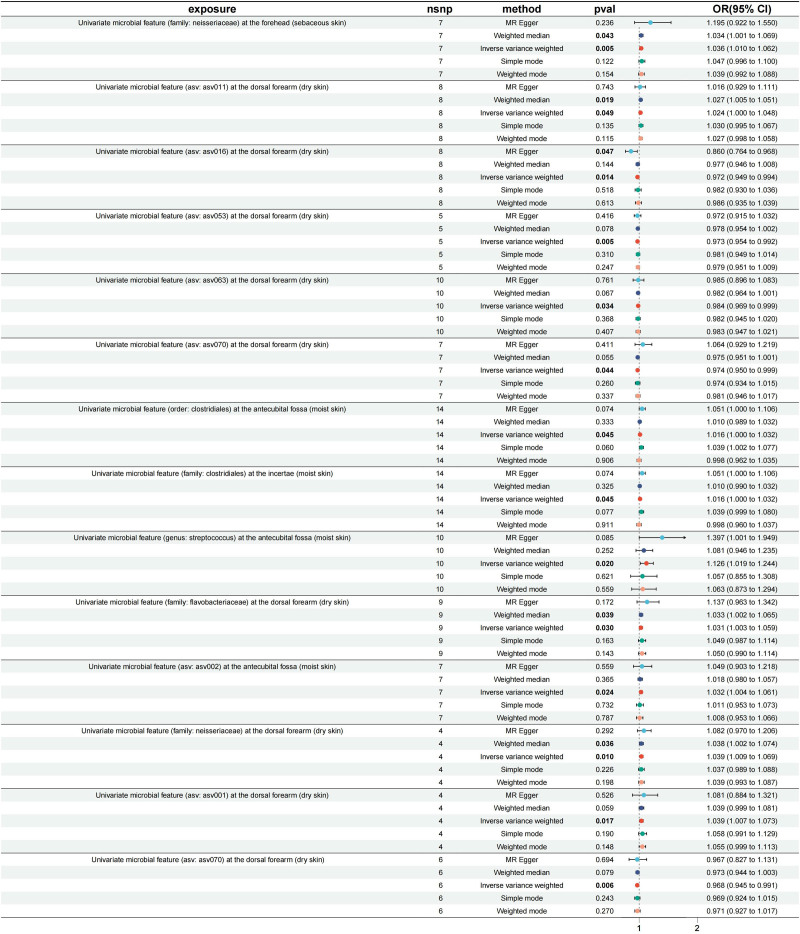
Forest plot displaying the results of the Mendelian randomization analysis, highlighting the positive association between skin microbiota (exposure) and psoriasis (outcome).

**Figure 3.**
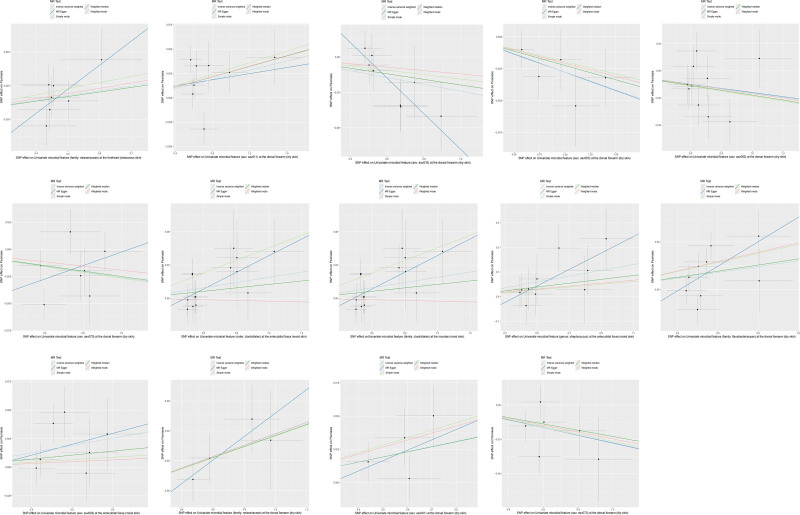
Scatter plot presenting the results of the positive Mendelian randomization analysis for psoriasis (exposure) and skin microbiota (outcome).

Mendelian randomization analysis revealed significant associations between several microbial features and psoriasis. Specifically, certain microbial features were identified as potential risk factors for psoriasis (Figs. 2 and 3). In the sebaceous skin of the forehead, the odds ratio (OR) for the family Neisseriaceae was 1.036 (95% confidence interval [CI]: 1.010–1.062, *P* = .0054). In the dry skin of the dorsal forearm, the OR for ASV011 was 1.024 (95% CI: 1.000–1.048, *P* = .0490). In the moist skin of the antecubital fossa, the OR for the order Clostridiales and the family Clostridiales were both 1.016 (95% CI: 1.000–1.032, *P* = .0449). Additionally, the OR for the genus Streptococcus in the antecubital fossa was 1.126 (95% CI: 1.019–1.244, *P* = .0196), for the family Flavobacteriaceae in the dorsal forearm was 1.031 (95% CI: 1.003–1.059, *P* = .0301), for ASV002 in the antecubital fossa was 1.032 (95% CI: 1.004–1.061, *P* = .0235), for the family Neisseriaceae in the dorsal forearm was 1.039 (95% CI: 1.009–1.069, *P* = .0095), and for ASV001 in the dorsal forearm was 1.039 (95% CI: 1.007–1.073, *P* = .0165; Figs. 2 and 3)

Conversely, some microbial features were identified as potential protective factors against psoriasis (Figs. 2 and 3). In the dry skin of the dorsal forearm, the OR for ASV016 was 0.972 (95% CI: 0.949–0.994, *P* = .0136), for ASV053 was 0.973 (95% CI: 0.954–0.992, *P* = .0054), for ASV063 was 0.984 (95% CI: 0.969–0.999, *P* = .0341), and for ASV070 was 0.974 (95% CI: 0.950–0.999, *P* = .0438). Additionally, the OR for ASV070 in the dorsal forearm was 0.968 (95% CI: 0.945–0.991, *P* = .0065; Figs. 2 and 3).

### 3.2. Reverse 2-sample Mendelian randomization study

Reverse Mendelian randomization analysis assessed the impact of psoriasis as an exposure on the skin microbiome. The results revealed significant associations between psoriasis and the skin microbiome in different skin regions.

**Figure 4.**
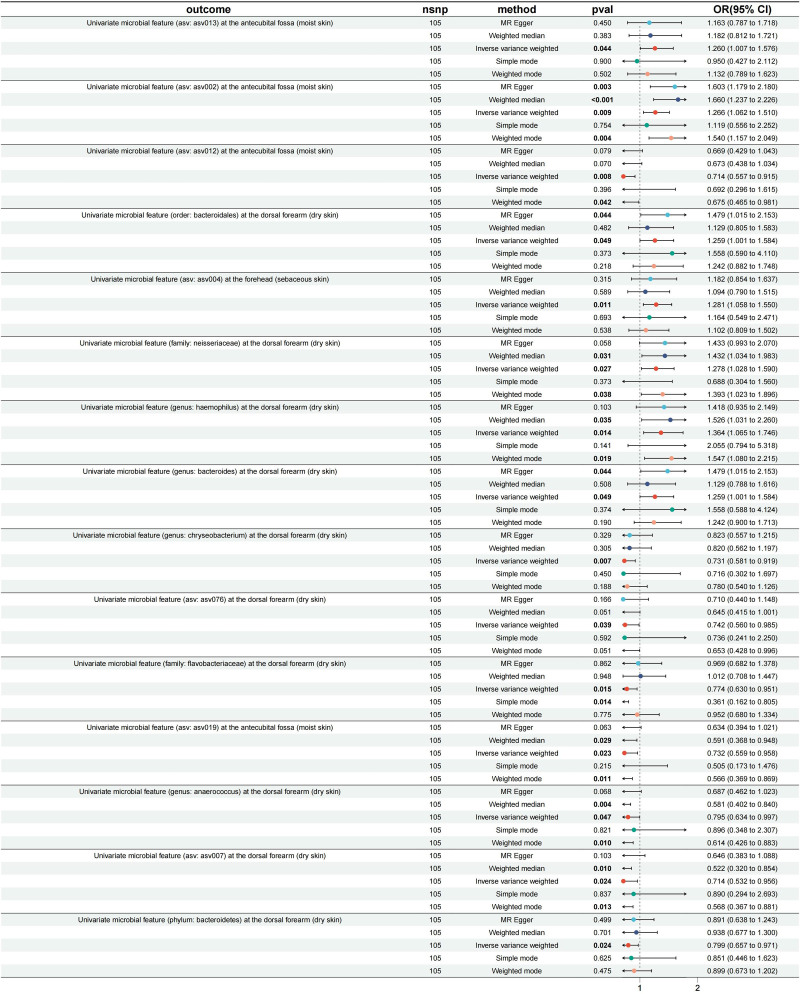
Forest plot of the reverse Mendelian randomization analysis, showing the relationship between psoriasis (exposure) and skin microbiota (outcome).

In the forearm (dry skin) region, psoriasis was identified as a significant risk factor (Fig. 4). Specifically, the OR for ‘asv002’ and ‘genus: Haemophilus’ were 1.266 (95% CI: 1.061–1.510, *P* = .027) and 1.364 (95% CI: 1.065–1.746, *P* = .013), respectively, indicating a strong association with psoriasis. Additionally, the OR for ‘genus: Bacteroides’ was 1.259 (95% CI: 1.000–1.584, *P* = .049), also showing a significant association. In the forehead (sebaceous skin) region, the OR for ‘asv004’ was 1.281 (95% CI: 1.058–1.549, *P* = .011), indicating its role as a risk factor for psoriasis (Fig. 4).

Conversely, in the forearm (dry skin) region, psoriasis showed a protective effect on certain microbial features (Fig. 4). The OR for ‘asv012’ was 0.714 (95% CI: 0.556–0.915, *P* = .007), for ‘asv076’ was 0.742 (95% CI: 0.559–0.990, *P* = .045), for ‘family: Flavobacteriaceae’ was 0.774 (95% CI: 0.591–0.998, *P* = .048), and for ‘phylum: Bacteroidetes’ was 0.799 (95% CI: 0.657–0.971, *P* = .024). These results suggest that these microbial features have a protective effect under the influence of psoriasis (Fig. 4).

We found that ‘asv002’ in the antecubital fossa (moist skin), ‘Neisseriaceae’ in the dorsal forearm (dry skin), and ‘Flavobacteriaceae’ in the dorsal forearm (dry skin) had bidirectional effects with psoriasis, while the remaining were unidirectional causal effects. Overall, this study explored the complex relationship between psoriasis and the microbiome of different skin regions, revealing the potential role of exposure-microbiome interactions in skin diseases. These findings provide important evidence for further research into the pathogenesis of psoriasis and the role of the microbiome in skin diseases.

### 3.3. Heterogeneity and pleiotropy tests

**Figure 5.**
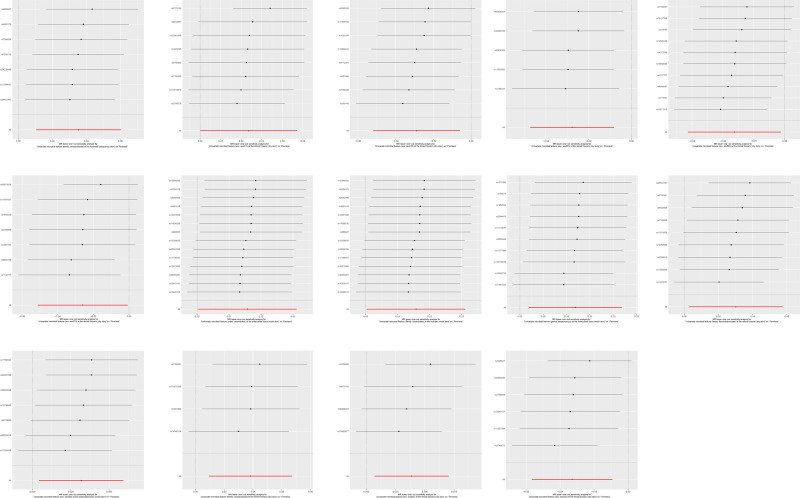
Leave-one-out analysis demonstrating the robustness of the results.

The complete results of heterogeneity and pleiotropy tests for forward and reverse MR analyses are presented in Tables 1 and 2. We performed Cochran’s *Q* test for the associations between 150 skin microbiome traits and psoriasis, with most *P* values >.05, indicating the absence of heterogeneity. Importantly, all *P* values for the MR-Egger intercepts were >.05, excluding bias due to horizontal pleiotropy. Additionally, the MR-PRESSO global test also indicated that the MR analysis results were not influenced by horizontal pleiotropy (*P* > .05). The results of the leave-one-out analysis are presented in Figure 5.

**Table 1:** Heterogeneity and pleiotropy results of dual sample positive analysis of skin microbiota and psoriasis.

Exposure	Method	Heterogeneity		Pleiotropy	
		*Q*	*Q*_pval	Intercept	*P*
Univariate microbial feature (family: neisseriaceae) at the forehead (sebaceous skin)	MR Egger	3.007	0.699	−0.067	.327
	Inverse variance weighted	4.184	0.652		
	PRESSO				.687
Univariate microbial feature (asv: asv011) at the dorsal forearm (dry skin)	MR Egger	13.876	0.031	0.006	.863
	Inverse variance weighted	13.951	0.052		
	PRESSO				.074
Univariate microbial feature (asv: asv016) at the dorsal forearm (dry skin)	MR Egger	1.779	0.939	0.073	.085
	Inverse variance weighted	6.02	0.537		
	PRESSO				.553
Univariate microbial feature (asv: asv053) at the dorsal forearm (dry skin)	MR Egger	1.278	0.734	0.001	.975
	Inverse variance weighted	1.28	0.865		
	PRESSO				.89
Univariate microbial feature (asv: asv063) at the dorsal forearm (dry skin)	MR Egger	10.14	0.255	−0.001	.982
	Inverse variance weighted	10.141	0.339		
	PRESSO				.349
Univariate microbial feature (asv: asv070) at the dorsal forearm (dry skin)	MR Egger	7.68	0.175	−0.065	.253
	Inverse variance weighted	10.242	0.115		
	PRESSO				.156
Univariate microbial feature (order: clostridiales) at the antecubital fossa (moist skin)	MR Egger	6.685	0.878	−0.022	.184
	Inverse variance weighted	8.673	0.797		
	PRESSO				.805
Univariate microbial feature (family: clostridiales) at the incertae (moist skin)	MR Egger	6.685	0.878	−0.022	.184
	Inverse variance weighted	8.673	0.797		
	PRESSO				.81
Univariate microbial feature (genus: streptococcus) at the antecubital fossa (moist skin)	MR Egger	3.097	0.928	−0.124	.221
	Inverse variance weighted	4.858	0.847		
	PRESSO				.853
Univariate microbial feature (family: flavobacteriaceae) at the dorsal forearm (dry skin)	MR Egger	11.815	0.107	−0.053	.277
	Inverse variance weighted	14.161	0.078		
	PRESSO				.087
Univariate microbial feature (asv: asv002) at the antecubital fossa (moist skin)	MR Egger	5.975	0.309	−0.009	.838
	Inverse variance weighted	6.03	0.42		
	PRESSO				.44
Univariate microbial feature (family: neisseriaceae) at the dorsal forearm (dry skin)	MR Egger	0.425	0.809	−0.027	.529
	Inverse variance weighted	0.994	0.803		
	PRESSO				
Univariate microbial feature (asv: asv001) at the dorsal forearm (dry skin)	MR Egger	2.496	0.287	−0.022	.734
	Inverse variance weighted	2.686	0.443		
	PRESSO				.494
Univariate microbial feature (asv: asv070) at the dorsal forearm (dry skin)	MR Egger	5.69	0.223	0.001	.992
	Inverse variance weighted	5.691	0.338		
	PRESSO				.372

MR = Mendelian randomization, PRESSO = Pleiotropy Residual Sum and Outlier.

**Table 2:** Heterogeneity and pleiotropy results of dual sample reverse analysis of skin microbiota and psoriasis.

Outcome	Method	Heterogeneity		Pleiotropy	
		*Q*	*Q*_pval	Intercept	*P*
Univariate microbial feature (asv: asv013) at the antecubital fossa (moist skin)	MR Egger	97.5633	0.6327	0.0105	.6245
	Inverse variance weighted	97.8043	0.6525		
	PRESSO				.699
Univariate microbial feature (asv: asv002) at the antecubital fossa (moist skin)	MR Egger	81.094	0.9455	−0.0307	.0694
	Inverse variance weighted	84.4601	0.9198		
	PRESSO				.896
Univariate microbial feature (asv: asv012) at the antecubital fossa (moist skin)	MR Egger	117.1862	0.1605	0.0083	.73
	Inverse variance weighted	117.3224	0.1755		
	PRESSO				.194
Univariate microbial feature (order: bacteroidales) at the dorsal forearm (dry skin)	MR Egger	112.3232	0.2492	−0.0226	.2928
	Inverse variance weighted	113.5425	0.2456		
	PRESSO				.26
Univariate microbial feature (asv: asv004) at the forehead (sebaceous skin)	MR Egger	99.9475	0.5668	0.0106	.5541
	Inverse variance weighted	100.2998	0.5844		
	PRESSO				.575
Univariate microbial feature (family: neisseriaceae) at the dorsal forearm (dry skin)	MR Egger	128.5928	0.0446	−0.0156	.4494
	Inverse variance weighted	129.3126	0.0469		
	PRESSO				.055
Univariate microbial feature (genus: haemophilus) at the dorsal forearm (dry skin)	MR Egger	108.7775	0.3294	−0.0053	.8196
	Inverse variance weighted	108.8327	0.3534		
	PRESSO				.377
Univariate microbial feature (genus: bacteroides) at the dorsal forearm (dry skin)	MR Egger	112.3232	0.2492	−0.0226	.2928
	Inverse variance weighted	113.5425	0.2456		
	PRESSO				.257
Univariate microbial feature (genus: chryseobacterium) at the dorsal forearm (dry skin)	MR Egger	76.349	0.9772	−0.0158	.4649
	Inverse variance weighted	76.8871	0.9786		
	PRESSO				.981
Univariate microbial feature (asv: asv076) at the dorsal forearm (dry skin)	MR Egger	108.304	0.341	0.0059	.8246
	Inverse variance weighted	108.3559	0.3654		
	PRESSO				.4
Univariate microbial feature (family: flavobacteriaceae) at the dorsal forearm (dry skin)	MR Egger	74.6637	0.984	−0.0299	.1255
	Inverse variance weighted	77.0492	0.9779		
	PRESSO				.973
Univariate microbial feature (asv: asv019) at the antecubital fossa (moist skin)	MR Egger	96.3865	0.6643	0.0184	.4745
	Inverse variance weighted	96.9017	0.6763		
	PRESSO				.675
Univariate microbial feature (genus: anaerococcus) at the dorsal forearm (dry skin)	MR Egger	98.1419	0.6169	0.0187	.3854
	Inverse variance weighted	98.9017	0.6229		
	PRESSO				.554
Univariate microbial feature (asv: asv007) at the dorsal forearm (dry skin)	MR Egger	106.1021	0.3973	0.0127	.6497
	Inverse variance weighted	106.3158	0.4186		
	PRESSO				.403
Univariate microbial feature (phylum: bacteroidetes) at the dorsal forearm (dry skin)	MR Egger	90.8322	0.7986	−0.0145	.4318
	Inverse variance weighted	91.4551	0.8053		
	PRESSO				.826

MR = Mendelian randomization, PRESSO = Pleiotropy Residual Sum and Outlier.

## 4. Discussion

This study represents one of the most comprehensive Mendelian randomization (MR) investigations to date, evaluating the causal relationship between the skin microbiome and psoriasis. Through forward and reverse 2-sample MR analyses, we uncovered significant associations between psoriasis and the skin microbiome. The forward MR analysis identified certain microbial features as potential risk factors for psoriasis, while others demonstrated protective effects. These findings suggest that the skin microbiome may play a crucial role in the onset and progression of psoriasis.

In the forward analysis, several microbial features were significantly associated with psoriasis in different skin regions (such as the forehead, forearm, and antecubital fossa). Specifically, microbial features such as the family Neisseriaceae, ASV011, the order Clostridiales, the genus Streptococcus, and the family Flavobacteriaceae showed higher odds ratios (OR) in psoriasis patients compared to healthy individuals. Conversely, microbial features like ASV016, ASV053, ASV063, and ASV070 exhibited lower ORs in psoriasis patients, indicating their potential protective role.

There are limited studies directly investigating the relationship between the genus Neisseria and psoriasis. However, some research has indicated an increased representation of Neisseria in the skin microbiome of psoriasis patients. Fyhrquist et al^[23]^ found an increase in Neisseria and Streptococcus in psoriasis-affected skin compared to healthy skin. Kayiran et al^[24]^ also reported an increased presence of Neisseria and Streptococcus in the scalps of psoriasis patients. Similarly, our study identified Neisseria as a strong risk factor in sebaceous skin of the forehead (*P* = .0054), suggesting that Neisseria may influence the skin’s microenvironment and contribute to psoriasis pathogenesis through interactions with sebaceous gland secretions or local immune responses.^[25]^

The antecubital fossa is characterized by skin folds that trap sweat and sebum, creating a moist and oily environment conducive to the growth of Streptococcus.^[26,27]^ Streptococcus may impact skin health by secreting toxins and other metabolites.^[28]^ Psoriasis patients often have compromised skin barrier function, which can be exacerbated by the proliferation of Staphylococcus aureus in psoriatic lesions. This pathogen produces toxins that further damage the skin barrier. Rachakonda et al^[11]^ demonstrated a significant association between Streptococcus infection and psoriasis, with Streptococcus infections frequently triggering acute psoriasis flares.^[11]^ The scratching induced by psoriasis-associated itching can further damage the skin, allowing bacteria (both commensals and pathogens) to penetrate deeper layers or even the bloodstream.^[29]^

There is currently limited research directly addressing the relationship between Clostridium species in moist skin and psoriasis, with most studies focusing on the gut microbiome. While Clostridium in the gut produces short-chain fatty acids that might influence systemic immune responses and potentially protect skin health, our findings suggest Clostridium as a risk factor for psoriasis.^[30]^ Further research is needed to clarify this relationship and explore the role of Clostridium in the skin microbiome.

These results support previous research linking the skin microbiome to psoriasis. Given the protective and harmful roles of these microbial features in psoriasis, future studies could explore therapies that supplement beneficial microbes or inhibit harmful ones. For instance, targeting areas with high sebum production and skin folds in psoriasis patients, along with monitoring the health of the microbiome, might be beneficial. Probiotic therapies or targeted antimicrobial treatments (e.g., against Neisseria, Streptococcus, and Clostridium) could represent new therapeutic strategies. Researching key microbial features and their interactions with the skin barrier could lead to personalized treatments based on the specific microbiome characteristics of psoriasis patients.

## Acknowledgments

We thank the authors in the article for their contributions.

## Author contributions

**Conceptualization:** Shangyi Xu, Kaiwen Yang, Jin Qiu, Jianqiao Zhong, Dehai Xian.

**Data curation:** Shangyi Xu, Kaiwen Yang, Jin Qiu, Jianqiao Zhong, Dehai Xian.

**Formal analysis:** Shangyi Xu, Kaiwen Yang, Jin Qiu, Jianqiao Zhong, Dehai Xian.

**Funding acquisition:** Shangyi Xu, Jianqiao Zhong, Dehai Xian.

**Investigation:** Kaiwen Yang, Dehai Xian.

**Methodology:** Dehai Xian.

**Project administration:** Kaiwen Yang, Dehai Xian.

**Resources:** Kaiwen Yang.

**Software:** Kaiwen Yang.

**Supervision:** Kaiwen Yang.

**Visualization:** Kaiwen Yang.

**Writing – original draft:** Kaiwen Yang.

**Writing – review & editing:** Kaiwen Yang.
